# Increased myocardial SERCA expression in early type 2 diabetes mellitus is insulin dependent: In vivo and in vitro data

**DOI:** 10.1186/1475-2840-11-57

**Published:** 2012-05-23

**Authors:** Sabine Fredersdorf, Christian Thumann, Wolfram H Zimmermann, Roland Vetter, Tobias Graf, Andreas Luchner, Günter AJ Riegger, Heribert Schunkert, Thomas Eschenhagen, Joachim Weil

**Affiliations:** 1 Klinik und Poliklinik für Innere Medizin II, Universität Regensburg, Regensburg, Germany; 2 Institut für Pharmakologie, Universitätsmedizin, Georg-August Universität Göttingen, Göttingen, Germany; 3 Institut für Klinische Pharmakologie und Toxikologie, Universitätsmedizin - Berlin, Berlin, Germany; 4 Medizinische Klinik II, Universitätsklinikum Schleswig-Holstein, Campus Lübeck, Lübeck, Germany; 5 Institut für Klinische und Experimentelle Pharmakologie und Toxikologie, Universität Hamburg, Hamburg, Germany; 6 Klinik und Poliklinik für Innere Medizin II des Universitätsklinikums Regensburg, 93042, Regensburg, Germany

**Keywords:** Diabetic heart, Insulin, SERCA expression, Relaxation velocity

## Abstract

**Background:**

Calcium (Ca2+) handling proteins are known to play a pivotal role in the pathophysiology of cardiomyopathy. However little is known about early changes in the diabetic heart and the impact of insulin treatment (Ins).

**Methods:**

*Zucker Diabetic Fatty* rats treated with or without insulin (ZDF ± Ins, n = 13) and lean littermates (controls, n = 7) were sacrificed at the age of 19 weeks. ZDF + Ins (n = 6) were treated with insulin for the last 6 weeks of life. Gene expression of Ca2+ ATPase in the cardiac sarcoplasmatic reticulum (SERCA2a, further abbreviated as SERCA) and phospholamban (PLB) were determined by northern blotting. Ca2+ transport of the sarcoplasmatic reticulum (SR) was assessed by oxalate-facilitated 45Ca-uptake in left ventricular homogenates. In addition, isolated neonatal cardiomyocytes were stimulated in cell culture with insulin, glucose or triiodthyronine (T3, positive control). mRNA expression of SERCA and PLB were measured by Taqman PCR. Furthermore, effects of insulin treatment on force of contraction and relaxation were evaluated by cardiomyocytes grown in a three-dimensional collagen matrix (engineered heart tissue, EHT) stimulated for 5 days by insulin. By western blot phosphorylations status of Akt was determed and the influence of wortmannin.

**Results:**

SERCA levels increased in both ZDF and ZDF + Ins compared to control (control 100 ± 6.2 vs. ZDF 152 ± 26.6* vs. ZDF + Ins 212 ± 18.5*# % of control, *p < 0.05 vs. control, #p < 0.05 vs. ZDF) whereas PLB was significantly decreased in ZDF and ZDF + Ins (control 100 ± 2.8 vs. ZDF 76.3 ± 13.5* vs. ZDF + Ins 79.4 ± 12.9* % of control, *p < 0.05 vs control). The increase in the SERCA/PLB ratio in ZDF and ZDF ± Ins was accompanied by enhanced Ca2+ uptake to the SR (control 1.58 ± 0.1 vs. ZDF 1.85 ± 0.06* vs. ZDF + Ins 2.03 ± 0.1* μg/mg/min, *p < 0.05 vs. control). Interestingly, there was a significant correlation between Ca2+ uptake and SERCA2a expression. As shown by in-vitro experiments, the effect of insulin on SERCA2a mRNA expression seemed to have a direct effect on cardiomyocytes. Furthermore, long-term treatment of engineered heart tissue with insulin increased the SERCA/PLB ratio and accelerated relaxation time. Akt was significantly phosphorylated by insulin. This effect could be abolished by wortmannin.

**Conclusion:**

The current data demonstrate that early type 2 diabetes is associated with an increase in the SERCA/PLB ratio and that insulin directly stimulates SERCA expression and relaxation velocity. These results underline the important role of insulin and calcium handling proteins in the cardiac adaptation process of type 2 diabetes mellitus contributing to cardiac remodeling and show the important role of PI3-kinase-Akt-SERCA2a signaling cascade.

## Introduction

The sarcoplasmatic reticulum (SR) plays a pivotal role in the contraction and relaxation cycle of the heart by virtue of its ability to tightly regulate intracellular calcium (Ca2+) concentration. Myocardial contraction is initiated by Ca2+ entry through Ca2+ channels of the plasma membrane (L-type Ca2+ channel) [1] triggering the Ca2+ release from the SR through the ryanodine receptor. During diastole Ca2+ is transported from the cytoplasma into the SR by the SR Ca2 + ATPase (SERCA). Recent cloning analysis revealed three distinct genes encoding for SR Ca2 + ATPases (SERCA 1–3), of which the SERCA2a is predominately expressed in cardiac tissue [2]. SERCA2a activity depends on the amount of SERCA2a protein and is further regulated by its inhibitory protein phospholamban (PLB) [3]. SERCA2a is the major determinant of the beat-to-beat regulation of cardiac contraction, and overexpression of this protein in mice has been shown to enhance myocardial relaxation [4]. Unphosphorylated PLB inhibits the Ca2+ uptake of SERCA2a and phosphorylation of the protein disrupts the inhibitory interaction resulting in increased Ca2+ transport towards the SR. Recent studies on PLB knockout mice have underlined the importance of PLB as a key regulator of cardiac contraction and relaxation [5].

While diabetes leads to cardiomyopathy in later stages with hypocontractility and reduced SERCA activity [6], we have observed increased contractility in earlier stages of type 2 diabetes mellitus [7]. Various animal models of diabetes have been used to study the causes and underlying subcellular events of diabetic cardiomyopathy. These studies suggest a dysfunctional sarcoplasmic reticulum (SR), leading to altered intracellular calcium handling in cardiac myocytes. This mechanism might be involved in the development of diabetic cardiomyopathy [6,8]. A reduced sequestration of calcium into the SR could readily explain the prolonged cardiac relaxation observed in diabetic cardiomyopathy. As a consequence, the SR calcium content declines, leading to a reduced systolic calcium release and therefore a weaker cardiac contraction. However, these studies were mainly carried out in models resembling insulin-dependent diabetes. Previous studies with diabetes type 1 models have shown a down-regulation of SERCA2a in the heart associated with a decrease in systolic and diastolic function. These functional alterations can be reversed by insulin treatment or SERCA2a overexpression [4,9]. So far, there is little information available on myocardial SERCA2a and PLB changes in the early stages of type 2 diabetes which is characterized by rather high insulin levels [8,10,11].

The *Zucker Diabetic Fatty* rat (ZDF/Drt-fa) is characterized by early onset of hyperglycemia, hyperphagia, hyperinsulinemia, adiposity and hyperlipidemia, thereby resembling the clinical features of human type 2 diabetes [12]. In an earlier study, we were able to demonstrate that animals in transition from insulin resistance to type 2 diabetes but not lean control rats develop significant myocardial hypertrophy associated with increased systolic function as evaluated by echocardiography [7]. These findings raised the possibility that one or more proteins regulating intracellular calcium homeostasis may be altered in these animals. Therefore, the present study was designed to determine whether the cardiac phenotype of ZDF rats is associated with alterations in cardiac SR function and expression of SERCA2a and its regulating protein PLB. Since insulin treatment is widely used as a therapeutic option for patients with poorly controlled type 2 diabetes, its impact on the aforementioned proteins was studied in isolated cell preparations of the heart. Furthermore, we investigated the functional consequences of high insulin levels in an innovative engineered heart tissue model.

## Materials and methods

### Animal model

Male *Zucker Diabetic Fatty* rats (body weight (BW) range: 106–158 g, n = 13) and male *Zucker* lean rats (BW 85–118 g, n = 7) were obtained at the age of five weeks from Genetic Models (Indianapolis, USA). Animals were maintained on RMH-B rat chow from Hope Farms (Woerden, Netherlands) with water ad libitum. All animals were individually housed in a 12 h dark/light cycle controlled room. The protocol had been approved by the local committee on animal research and conforms to the *Guide for the Care and Use of Laboratory Animals*, published by the US National Institutes of Health (NIH Publication No. 85–23; revised 1985). At the age of 13 weeks, one week after developing hyperglycemia, the animals were divided into three groups: (1) *Zucker* lean rats (control group, n = 7), (2) *Zucker Diabetic Fatty* rats without insulin treatment (ZDF; n = 7), and (3) *Zucker Diabetic Fatty* rats treated with insulin (ZDF + Ins; n = 6). Insulin treatment (Actrapid HM U500, Novo Nordisk, Mainz, Germany) was initiated at a dose of 25.0.

U/kg/day with subcutaneously implanted Alzet osmotic minipumps (Model 2ML2 and 2ML4, Charles River Wiga, Sulzfeld, Germany). Pumps were changed after 2 weeks and the insulin dose was adapted to normalize blood glucose levels. Body weight was determined every week, and blood glucose levels every 2–3 weeks (Accu-Chek Plus Roche, Mannheim, Germany). At the age of 18 weeks, systolic blood pressure and heart rate were measured by indirect tail-cuff method as described [13] using an automated cuff inflator-pulse detection system (W + W electronic AG, BP recorder No. 8005, Basel, Switzerland). After 6 weeks of insulin treatment, at the age of 19 weeks, the animals were killed.

### Tissue preparation

Hearts were rapidly excised, rinsed with saline and blotted dry. The whole heart weight was determined. The heart was dissected free from the atria, cut into right and left ventricular tissue, frozen in liquid nitrogen within 3 minutes and stored at −80 C until analyzed, as described earlier [7].

### Neonatal rat cardiac myocytes

Rat cardiac myocytes were isolated from 1- to 3-day-old neonatal Wistar rats (University of Regensburg breed, from Charles River, Sulzfeld, Germany) as described earlier [14]. Briefly, hearts from 50–70 pups were minced and subjected to serial trypsin digestion to release single cells. After the final digestion, cells were washed and pre-plated for 1–2 h in complete culture medium (MEM supplemented with 10% fetal calf serum and 1% penicillin/streptomycin). Unattached cells were pelleted and suspended in culture medium containing 0.1 mmol/l 5´-bromo-2´-desoxyuridine (BrdU) to suppress overgrowth of non- myoctes. Cells were then plated on culture dishes at a density of 150,000 cells/cm2 and incubated for 5 days at 37°C before being stimulated with insulin (0.1-3.0 μmol/l), wortmannin (300 and 1000 nM) or triiodothyronine (3.0 nmol/l) as a positive control for up-regulation of SERCA2a mRNA expression [15].

### Engineered heart tissues

Engineered heart tissues (EHT) were prepared as described previously [16]. Briefly, circular EHT were prepared by mixing freshly isolated cardiac myocytes from neonatal rats with collagen type 1 prepared from rat tails, a basement membrane mixture (Matrigel, tebu, Offenbach, Heidelberg, Germany), and concentrated serum containing culture medium (2xDMEM, 20% horse serum, 4% chicken embryo extract, 200 U/ml penicillin and 200 μg/ml streptomycin); pH was neutralized by titration with NaOH (0,1 N). The reconstitution mix was pipetted into circular casting molds and incubated for 30 to 45 min at 37oC and 5% CO2 to allow hardening of the reconstitution mix. Thereafter, 5 ml serum-containing culture medium (DMEM, 10% horse serum, 2% chicken embryo extract, 100 U/ml penicillin and 100 μg/ml streptomycin) was added to each dish. Culture was performed as described earlier [16]. After 7 days in culture, EHTs were transferred to a stretch device and subjected to phasic stretch (to 110% of their original length) for 5 days. Culture medium was changed 12 hours after EHT casting and then every other day. After transfer to the stretch device, the culture medium was changed every day and supplemented with insulin at a high physiological concentration (0.1 μg/ml; Sigma-Aldrich, Taufkirchen, Germany).

### Force measurement

After 12 days (7 days in casting molds and 5 days of stretching), the EHTs were transferred to thermostated organ baths containing gassed Tyrode´s solution and subjected to isometric force measurement as described elsewhere [16]. Briefly, electrically stimulated EHTs (2 Hz) were stretched to the length at which force of contraction was maximal and inotropic and lusitropic responses to cumulative concentrations of isoprenaline (0.1-1000 nM) in the presence of 0.2 mM calcium were recorded. Contractile activity was evaluated with a PC-assisted system (BMON2, Ingenieurbüro Jäckel, Hanau, Germany).

### RNA analysis

Total RNA from left ventricles or cultured cardiac myocytes was isolated with Trizol® (Canadian Life Technologies Inc., Burlington, Ontario, Canada) according to the manufacturer’s instructions. The concentration was determined photometrically at 260 nm. Total RNA was stored at 80°C. For Northern blot analysis 20 μg of total RNA were denatured, size-fractionated by electrophoresis on 1% agarose gels under denaturing conditions, transferred to nylon membranes (Gene Screen Plus, NEN, Dreieich, Germany) and immobilized by ultraviolet irradiation. Blots were prehybridized and hybridized using standard protocols as described previously [17]. Hybridized filters were washed and exposed at −80 C° to x-ray films (XAR-5, Eastman Kodak, N.Y., USA) by using intensifying screens. Different exposures of all autoradiograms were obtained to ensure that laser scanning (Personal Densitometer No. 50301, Molecular Dynamics) was performed within the linear range of densitometry. For hybridization cDNA probes for rat SERCA, PLB (kindly gifted by K.R. Boeheler) and GAPDH were radiolabelled with α32-P dCTP (specific activity 3000 Ci/mmol, Amersham, Dreieich, Germany) for Northern blot analysis. Values were normalized to these house-keeping gene GAPDH. The rat cDNA of GAPDH was cloned by reverse transcriptase PCR using the following primers: forward 5´-CTTCACCACCATGGAGAAGG-3´; and reverse 5´-ATTGAGAGCAATGCCAGCC-3´.

For quantitative RT-PCR (qRT-PCR), total RNA was transcribed with SuperScriptII RT (Invitrogen, CA, USA). Individual samples of 20 ng cDNA were amplified with AmpliTaqGold Polymerase (Applied Biosystems, CA, USA) utilizing gene specific primers and fluorogenic probes (5’ FAM and 3’ TAMRA; see below for complete primer/probe sequence information) in an ABI PRISM® 7900HT Sequence Detection System (Applied Biosystems). Probes were designed to cross exon/intron boundaries with primer annealing sites being located in the adjacent exons to eliminate the possibility of genomic DNA amplification. . Standard curves were performed in duplicate with serially diluted cDNA from neonatal rat heart tissue (1.5 - 50 ng) to determine PCR efficiency, which was similar in all groups SERCA and Phospholamban expression were evaluated as SERCA/PLB ratio and correlated to EHT twitch tension and relaxation time (T2). Quantification was performed by the standard curve and 2-ΔΔCt methods [18].

SERCA2a: forward primer 5´- AGT GGC TGA TGG TGC TGA AA-3´.

reverse primer 5´- GCA CCC GAA CAC CCT TAC AT-3.

probe 5´ FAM- TTA CTC CAG TAT TGC AGG CTC CAG GTA -TAMRA 3´.

PLB: forward primer 5´- GCA GCT GAG CTC CCA GAC TT-3.

reverse primer 5´- TTT CCA TGA TGC CAG GAA GAC-3´.

probe 5´ FAM- CAC AGA AGC CAA GGC CTC CTA AAA GGA G -TAMRA 3´.

We checked 18 S, GAPDH, and CSQ2 (data not shown). However, corrections are not necessary because we have determined PLB and SERCA from the same cDNA samples.

### Western blot analysis

20 μl of cell suspension of the cardiomyocyte cell culture were separated on 10% SDS-polyacrylamide gels. Gels were run andseparated proteins were transferred to nitrocellulose membranes in 50 mM sodium phosphate buffer, pH 7.4, for 20 h at 300 mA, and 4°C. Nitrocellulose sheets were incubated with a rabbit polyclonal anti-human antisera (Sigma) at a 1:2000 dilution. Phospho-Akt and Akt antibodies (1:1000, from New England Biolabs) were visualized colorimetrically by using horseradish peroxidase- (HRP) conjugated goat anti-rabbit immunoglobulin G at a 1:1000 dilution. After phospho-Akt blotting, the blot was stripped for 30 min at 50oC and then blotted for Akt, serving also as loading control. Apparent molecular weights were determined by using a prestained standard (kaleidoscope prestained standard, Biorad, USA).

### Oxalate-supported Ca2+ uptake

Oxalate-supported SR Ca2+ uptake was measured in left ventricular homogenates as described previously [19]. Briefly, the Ca2+ uptake medium of 0.2 ml contained 40 mmol/l imidazole (pH 7.0), 100 mmol/l KCl, 5 mmol/l MgCl2, 5 mmol/l TrisATP, 6 mmol/l phosphocreatine, 10 mmol/l K + -oxalate, 10 mmol/l NaN3, 10 μM synthetic protein kinase A-inhibitor peptide [PKI(6–22)amide; GIBCO-BRL, Eggenstein, Germany], 0.2 mmol/l EGTA, and 0.08 or 0.250 mmol/l 45CaCl2 corresponding to 0.34 or 3.68 μmol/l free Ca2+, respectively After 2 min of preincubation at 37°C, the measurement was started by addition of homogenate (30 μg protein) and 2 min later a 0.15-ml sample was filtered through 0.45- μm Millipore filters using a vacuum pump. The filter was immediately washed twice with 3 ml ice-cold solution containing 100 mM KCl, 2 mM EGTA, and 40 mM imidazole (pH 7.0). Radioactivity bound to dry filters was determined by liquid scintillation counting. All measurements were done in duplicate Ca uptake was measured within the linear range of the reaction. Calculated Ca2+ uptake values were expressed as nmoles of Ca2+ per mg of protein per min or μmoles of Ca2+ per g wet LV wt min.

### Statistics

Statistical analysis was performed using GraphPad PRIZM 5.0. Results are expressed as mean ± SEM. Comparisons between multiple groups were assessed by one-way analysis ANOVA-test and post-hoc analysis by Bonferroni. The strength of the relationship between two variables was assessed by calculating the product–moment correlation coefficient *r*. Statistical significance was accepted at p < 0.05.

## Results

### Diabetic animals

The ZDF rats developed a manifest diabetes at the age of 12 weeks (Capillary Glucose control group 78 ± 1.8 mg/dl vs. ZDF 252 ± 39* mg/dl vs. ZDF + Ins 292 ± 33* g/dl*, p < 0.05 vs. control). Body weight in ZDF rats increased steadily over time compared to non-diabetic lean animals. Treatment with insulin led to a further increase in body weight three weeks after beginning treatment (see Table 1). Absolute heart weight was significantly higher in the treatment group, but not in non-treated ZDF rats compared to age- matched ZDF rats (see Table 1). As expected, treatment with insulin decreased blood glucose level towards normoglycemic values in diabetic animals (see Table 1). Plasma C-peptide-levels were markedly elevated in diabetic ZDF rats indicating severe hyperinsulinemia. Interestingly, heart rate was significantly lower in all diabetic animals and blood pressure was the same in the non-treated ZDF group and even reduced in the insulin-treated ZDF group (at 19 weeks of age: control 484± min-1 vs. ZDF 416 ± 13* min-1 vs. ZDF + Ins 421 ± 18* min-1, *p < 0.05 vs. control).

**Table 1:** Biometric data at 19 weeks

**Characteristics**	**Control (n = 7)**	**ZDF (n = 7)**	**ZDF + Ins (n = 6)**
BW (g)	353 ± 11	402 ± 9^*^	478 ± 25*
HW (mg)	1372 ± 33	1341 ± 46	1530 ± 53#
Rel. HW (mg/g BW)	4.1 ± 0.2	3.3 ± 0.1*	3.2 ± 0.4*
Serum glucose	96 ± 7	477 ± 26*	251 ± 73*
C-peptide (pmol/l)	593 ± 98	913 ± 65*	639 ± 162^#^
Hb1Ac (% of control)	100 ± 4	301 ± 12*	179 ± 19*#

ZDF = *Zucker Diabetic Fatty* rats, Ins = insulin, BW = body weight, HW = heart weight.

*p < 0.05 vs control and #p < 0.05 vs. ZDF.

### Expression levels of SERCA and phospholamban (PLB)

**Figure 1:**
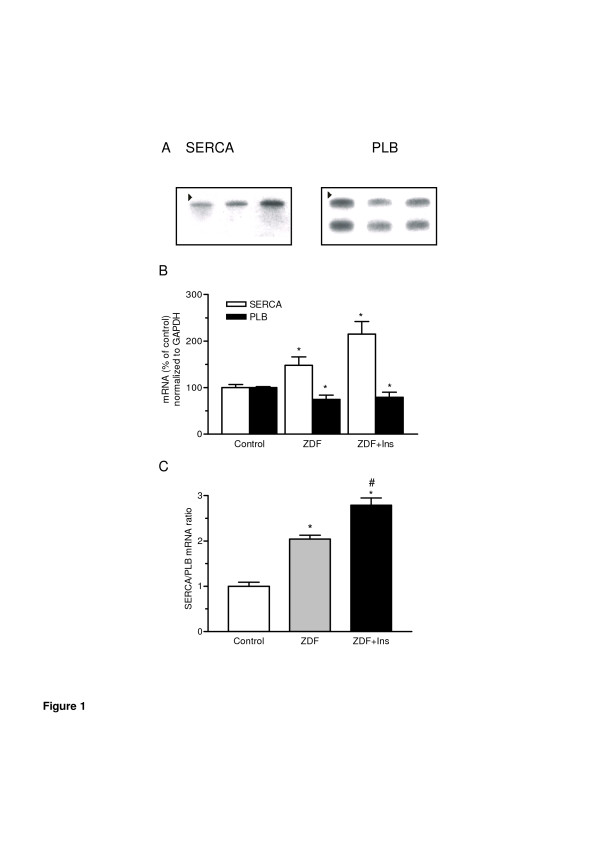
**In vivo data: Expression of SERCA 2a and PLB in left ventricular tissue from non-diabetic control rats (n = 7),*Zucker*Diabetes Fatty (ZDF) rats (n = 7) and ZDF (n = 6) rats treated with insulin.** (**A**) Shows a representative Northern blot of SERCA and phospholamban (lane 1 = left ventricular tissue from control rats, lane 2 = left ventricular tissue from diabetic ZDF rats; lane 3 = left ventricular tissue from ZDF rats treated with insulin). Arrow depicts the position of the 28 S ribosomal RNA. (**B**) quantitative analysis and (**C**) SERCA2a/PLB ratio. Values are given in mean ± SEM. *p < 0.05 vs. control; #p < 0.05 vs. ZDF.

**Figure 2:**
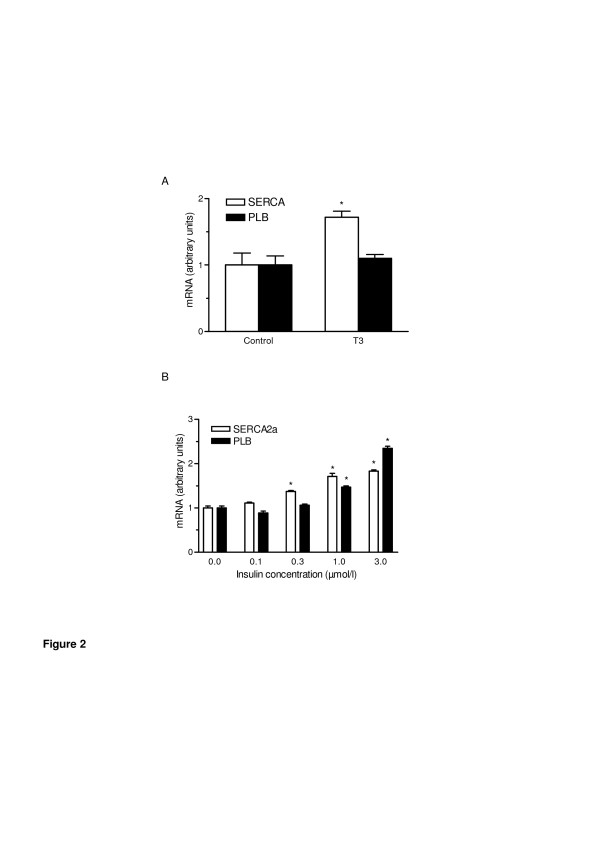
**In vitro data: (A) Effect of triiodothyronine (T3; 3.0 nmol/l) and (B) increasing concentrations of insulin on SERCA2a and PLB mRNA expression in isolated cardiac myocytes assessed by RT-PCR. T3 was used as positive control.** N = 9 in each group from three independent experiments. Values are given as mean ± SEM. *p < 0.05 vs. control (**A**), *p < 0.05 vs. no insulin (**B**).

SERCA2a detected a single mRNA of about 4.0 kb. The PLB probe hybridized with two mRNA species of approximately 3.0 and 1.3 kb (Figure 1A). As shown in Figure 1B SERCA2a mRNA levels were significantly higher in diabetic compared to non-diabetic animals and showed a trend towards a further in crease in insulin-treated animals (both p < 0.05 vs control). In contrast PLB mRNA levels were significantly reduced both in ZDF and ZDF + Ins (both p < 0.05 vs control). This led to an increase in the relative SERCA2a/PLB ratio (Figure 1c), indicating a facilitated intracellular calcium reuptake. Interestingly, treatment with insulin led to a further increase in SERCA2a mRNA in diabetic animals. To determine whether insulin directly up-regulates SERCA2a mRNA, isolated cardiac myocytes were treated with insulin over 5 days. As expected triiodothyronine (positive control) increased SERCA2a mRNA levels by approximately 75% as assessed by quantitative RT-PCR and had no effect on PLB expression (Figure 2A). Insulin led to a concentration-dependent increase in SERCA2a and PLB Fredersdorf et al. - Expression of SR Ca++ − uptake regulating proteins in the diabetic heart 11 expression (Figure 2B). Interestingly, SERCA2a expression was already increased at lower insulin concentrations (0.1–0.3 μmol/L) whereas PLB was unchanged. However, at higher insulin concentrations we found a similar increase in SERCA2a and PLB expression.

### Ca2 + −transport of myocardial sarcoplasmatic reticulum

**Figure 3:**
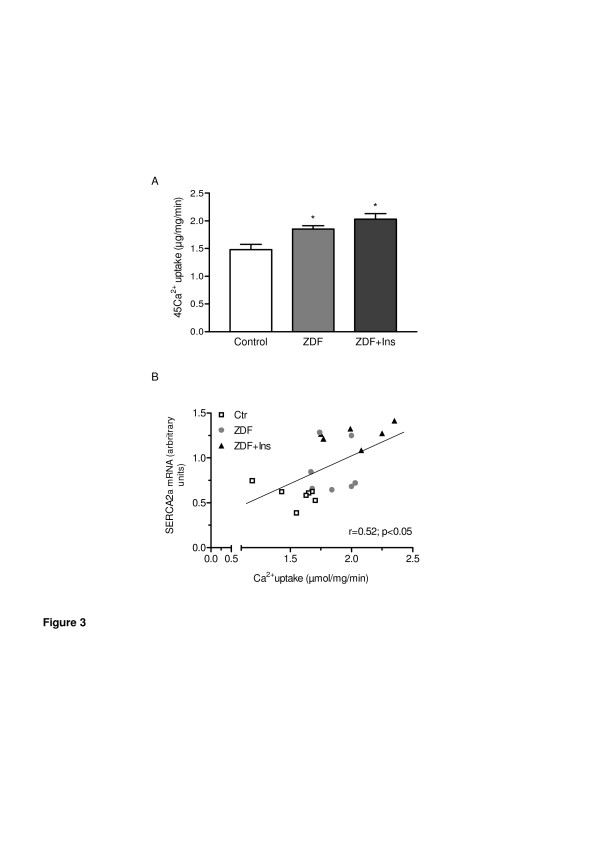
**In vivo data: Differences in sarcoplasmic reticular Ca2+ uptake in left ventricular homogenates prepared from (A) non-diabetic control rats (control) (n = 7),*Zucker*Diabetes Fatty (ZDF) rats (n = 7) and ZDF rats treated with insulin (ZDF + Ins, n = 6).** (**B**) Relationship between calcium uptake and myocardial SERCA2a/PLB mRNA ratio. Ca2+ uptake was measured for 2 min in the presence of 3.68 μM free Ca2+ concentrations. Values are given as mean ± SEM. *p < 0.05 vs. control.

To test whether the aforementioned changes in SERCA2a and PLB affect Ca2+ uptake of the sarcoplasmatic reticulum we measured oxalate-supported Ca2+ uptake in homogenates from left ventricular tissue of diabetic and non-diabetic animals. Ca2+ uptake was significantly higher in diabetic animals compared to non-diabetic animals (Figure 3A). Treatment with insulin further augmented Ca2+ uptake. As shown in Figure 3B there was a close relationship between myocardial SERCA2a mRNA expression and the calcium transport rate supporting the notion that insulin directly modifies SERCA2a mRNA expression in the heart.

### Effect of insulin on myocardial function in vitro

**Figure 4:**
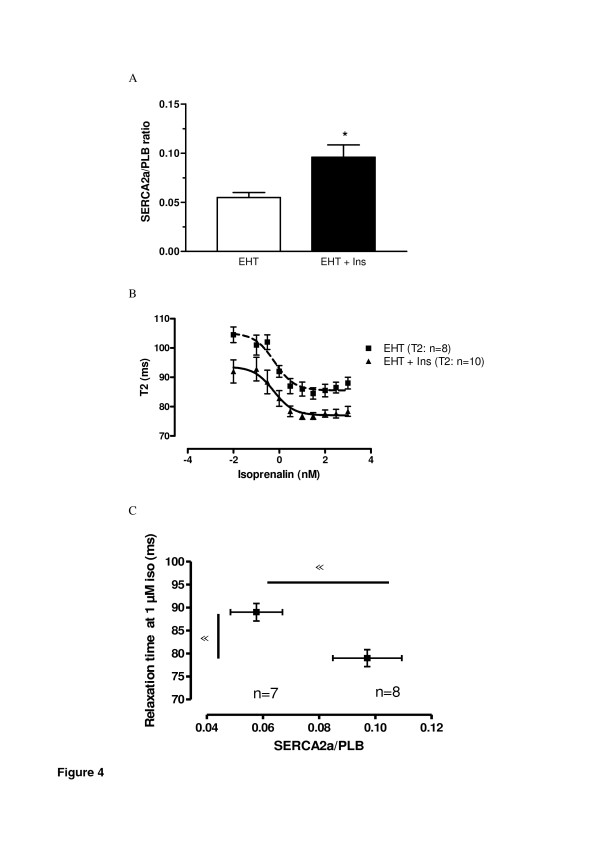
**In vitro data: Effect of insulin on SERCA2a/PLB mRNA ratio (A) and (B) EHT relaxation data (T2).** EHT were stimulated for 5 days with 0.10 μg/mL insulin over 5 days (n = 7-10) in each group from two independent cell preparations. Transcript concentration was determined by mRNA was measured by quantitative RT-PCR. *p < 0.01 vs. EHT. (**C**) Correlation between relaxation time and SERCA2a/PLB ratio in EHT´s without and with prior stimulation of insulin. Contractile response of isoprenaline (1 μmol/L) was determined under isometric conditions and in the presence of 0.2 mmol/L calcium (n = 7-8 in each group from two independent cell preparations). For clarity, only mean values ± SEM of the two groups are shown. *p < 0.05 vs. non insulin treated EHTs. In (**B**) relaxation time (T2) was significantly shorter in insulin stimulated EHTs (p < 0.05).

Engineered heart tissue (EHT) chronically stimulated (5 days) with insulin in a supra-physiological concentration (0.1 μg/mL) revealed a significant increase in the SERCA2a/PLB ratio as determined by quantitative RT-PCR (Figure 4 A).

To determine the influence of the observed changes in the SERCA2a/PLB ratio on myocardial function contractile parameters of electrically driven control EHTs and insulin-stimulated EHTs were measured. Isometric force development at optimal length in the absence (0.75 ± 0.12 vs. 0.78 ± 0.11 mN, n = 7 + 10; p = NS) and presence (1.32 ± 0.13 vs. 1.42 ± 0.08 mN, n = 7 + 10; p = NS) of isoprenaline (1 μmol/L) was not significantly different between control EHTs and insulin-stimulated EHTs (EHT 1.32 ± 0.13 vs. EHT + Ins 1.4 ± 0.14 mN p = NS). Interestingly, EHT relaxation (T2) was shorter in insulin treated EHTs (sustained positive lusitropic effect) under baseline conditions (EHT 105 ± 3 ms vs. EHT + Ins 92 ± 4 ms, n = 8-10; p < 0.05 vs. EHT) and under maximal isoprenaline stimulation (EHT 88 ± 2 ms vs. EHT + Ins 78 ± 2 ms, n = 8-10; p < 0.05 vs EHT; Figure 4B). In contrast, time to peak contraction was not affected by chronic insulin stimulation (data not shown).

Ultimately, the sustained lusitropic effect of insulin of isoprenaline as depicted in Figure 4B was considerably enhanced in EHTs treated with insulin compared to control EHTs. Interestingly, there was a significant correlation between the isoprenaline induced relaxation and the higher SERCA2a/PLB ratio (Figure 4C). The latter was the result of a higher SERCA2a concentration and at the same time lower PLB transcript concentration Notably, absolute PLB transcript abundance was markedly lower in insulin treated EHTs when compared to control EHTs.

### Effect of insulin on akt

**Figure 5:**
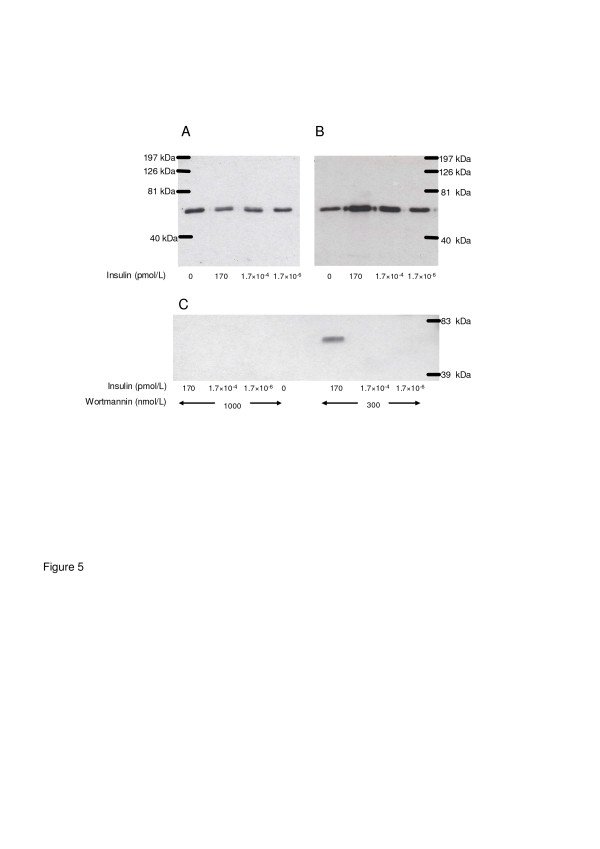
**Effect of increasing concentrations of insulin on the phosphorylation status of Akt in isolated cardiomyocytes.** (**A**) Western blot incubated with a specific antibody directed to total Akt. (**B**) The identical Western blot incubated with a specific antibody directed to phospho-Akt (Ser473). The insulin- mediated increase of phosphorylated Akt was abolished by the administration of an intermediated concentration of the PI3K-Inhibitor Wortmaninn, where Wortmaninn per se had no effect on phospho-Akt (**C**).

As shown by Figure 5, Akt was significantly phosphorylated by insulin (B) in cardiomyocytes. Unphosphorylated Akt blot served as control (A). This effect could be abolished by wortmannin (C) at higher wortmannin concentrations (1000 nM) for every insulin stimulation, at lower wortmannin concentration (300 nM) only for lower insulin concentrations.

## Discussion

The key findings of this study are that in the early stages of type 2 diabetes, myocardial expression of SERCA2a is markedly elevated, whereas the expression of PLB mRNA is reduced. These changes go along with a significant increase in sarcoplasmatic Ca2+ uptake. Interestingly, the SERCA/PLB ratio in diabetic animals was further increased by insulin treatment. In vitro, we were able to demonstrate that insulin treatment of isolated cardiac myocytes led to a concentration-dependent increase in SERCA2a expression. This effect was also seen in a more complex model of engineered heart tissue and correlated positively with cardiac relaxation in vitro. . In addition, Akt was significantly stimulated by insulin in cardiomyocyte cell culture. This approach allows us to directly determine contractile alterations caused by insulin treatment without considering insulin-related systemic changes which might also affect the cardiovascular system. However we must consider that in vitro data can not totally be compared to in vivo data. Nevertheless they give essential additional information. Together, this suggests that insulin, which is particularly elevated in premature type 2 diabetes mellitus, may be involved in the induction of SERCA2a mRNA expression and may be an early step in the pathogenesis of diabetic cardiomyopathy.

### Alterations of SERCA, PLB and ca uptake in diabetes mellitus

Diastolic relaxation of the heart is mediated to a large extent by the uptake of Ca2+ into the sarcoplasmic reticulum. Several studies have shown that diabetic cardiomyopathy is associated with decreased contractility and impaired relaxation [19]. These changes have been attributed to a reduced ability to sequester calcium into the SR, which primarily determines the speed of cardiac relaxation. Consecutive experiments have shown that SERCA2a mRNA expression, protein level and activity is down-regulated in streptozotocin-induced (STZ) type 1 diabetes mellitus [10,11]. In another model Wold and co-workers demonstrated in cardiomyocytes of rats with sucrose-induced insulin resistance that impaired SERCA activity with normal protein content contributes to cardiomyocyte dysfunction, whereas NCX function and expression are normal [20]. The authors concluded that subtle changes in Ca2+ regulation which occur prior to overt ventricular dysfunction and/or failure, may be common to early stages of a number of disorders involving insulin resistance. Furthermore, it has been demonstrated that reduced Ca2+ signaling in vascular smooth muscle cells from diabetic animals is related to a decline and/or redistribution in the IP3R Ca2+ channels and SERCA proteins. These changes could be repeated in cell culture experiments with high glucose levels [21]. The mechanism of alteration of SR proteins in STZ- induced diabetes, however, is unclear at present. The same is true for the *Otsuka-Long-Evans Tokushima Fatty* rats (OLETF), a type 2 diabetes model. Here in late stages of diabetes, after additional sucrose feeding, the ventricular relaxation rate was significantly slower and was associated with reduced SERCA2a level [22]. These experiments are therefore in apparent contrast to our results. This discrepancy is most likely due to the different models used and the rather advanced duration of the diabetes and older age (60 weeks in the latter experiments as compared to 19-week-old diabetic rats in our study). OLETF rats present a milder form of diabetes mellitus with later onset and milder hyperglycemia at the beginning [23]. We examined ZDF rats at earlier diabetic stages, and our previous experiments have shown that C-peptide levels are high at this age [7].

### Insulin directly up-regulates SERCA and preserves cardiac function

Most of the previous studies used a model with type 1 diabetes which differs fundamentally in pathophysiology. Whereas type 1 diabetes mellitus results from selective destruction of the insulin- producing beta cells of the pancreas, type 2 diabetes is primarily characterized by insulin resistance followed by progressive beta-cell dysfunction, resulting in low insulin levels in the long term. As recently shown, obese ZDF rats are insulin-resistant and have basal hyperinsulinemia that is due mainly to hypersecretion of insulin, as indicated by their elevated basal C-peptide levels [7,24]. Obese pre- diabetic and diabetic rats also show a reduction in insulin clearance, as indicated by their lower C- peptide/insulin ratio. Interestingly, the decrease in SERCA2a activity in STZ-treated rats can be reversed by insulin treatment [9,11], suggesting a direct stimulatory effect of insulin on SERCA2a. This hypothesis is further supported by other experiments demonstrating an up-regulation of SERCA1 in skeletal muscle after stimulation with insulin [1]. These observations demonstrate a possible link between insulin and expression of SR calcium ATPase, which is further confirmed by the present in vitro studies showing a direct effect of insulin on SERCA2a transcription in isolated cardiac myocytes. The important role of insulin for heart function is further supported by Kim et al., who showed that insulin preserves heart function in streptozotocin-induced diabetic heart failure with and without transplantation of smooth muscle cells [25].

In this context it has been shown that transgenic (TG) mice with cardiac-specific overexpression of active Akt not only exhibit hypertrophy and enhanced left ventricular function but also show a 6.6-fold increase in SERCA2a protein levels, which could be recapitulated in vitro by adenovirus-mediated overexpression of Akt in cultured adult ventricular myocytes [26]. We demonstrated on isolated cardiac myocytes a strong and rapid phosphorylation of Akt after stimulation with insulin. Conversely, inhibiting SERCA2a with either ryanodine or thapsigargin affected myocyte contraction and relaxation and Ca2+ channel kinetics more in TG than in WT. Thus, myocytes from mice with overexpressed Akt demonstrated enhanced contractility and relaxation, Fura-2 Ca2+ transients, and Ca2+ channel currents [26]. Furthermore, increased protein expression of SERCA2a plays an important role in mediating enhanced left ventricular function by Akt. Interestingly, insulin stimulation led to a significant increase in SERCA2a, co-immunoprecipitated with insulin receptor substrate proteins (IRS-1 and IRS-1) in isolated cardiac muscle demonstrating a link between insulin, insulin receptor and SERCA2a [27] in cardiac tissue.

Recent experiments showed that insulin-like growth factor 1 (IGF-1) activates multiple signaling pathways, which involve the activation of the phosphatidylinositol (PI)3-kinase and Akt [28]. It is well known that the PI3-kinase-Akt cascade modulates diverse cellular functions. Furthermore, it has been shown that that IGF-I caused increases in myocyte contraction and relaxation function, increases in intracellular Ca2+ transients, and an upregulation of SERCA2a [29]. The same group demonstrated that transgenic mice with cardiac-specific overexpression of Akt showed an enhanced left ventricular function, associated with an increased expression of SERCA2a [30]. These data are in accord with the studies of von Lewinski and co-workers [31](4), who suggested that Akt contribute to the acute inotropic effect of IGF-I in myocytes from human failing hearts.

In the present study, the insulin induced increase in phosphorylated Akt in isolated cardiomyocytes was abolished by the PI3-kinase inhibitor wortmannin, which provided evidence for a role of PI3-kinase. This finding suggests that the underlying cellular mechanism for up regulation of SERCA2a is mediated by the PI3-kinase-Akt-SERCA2a signaling cascade.

From a pathophysiological point of view, insulin-induced up-regulation of myocardial SERCA2a may be seen as a feedback mechanism in handling the volume overload caused by high glucose levels in the early phase of type 2 diabetes, when insulin levels are high. With progression of the disease and decreasing levels of insulin the expression of SERCA2a in the heart becomes impaired. The reduction of SERCA2a, as typically seen in the late phase of type 2 diabetes, is a major cause of reduced diastolic and systolic function of the heart. This hypothesis is reinforced by the findings of Sakata et al. who demonstrated that cardiac SERCA2a gene transfer restores systolic and diastolic function to normal in diabetic rats [8]. Furthermore, in-vitro experiments provide evidence that high glucose levels also impair cytosolic Ca2+ removal involving slowed SR Ca2+ uptake. It has been speculated that slowed SR Ca2+ uptake results from depressed protein kinase A (PKA) down-regulating SERCA2a, rather than through depressed SERCA expression. Both expression and function of the Na-Ca-exchanger (NCX) appear to be normal in these experiments [16]. In summary, the up-regulation of SERCA2a in the early phase of type 2 diabetes is an important physiological adaptation of the heart allowing it to handle volume overload caused by high glucose levels.

## Competing interests

The authors declare that they have no conflict of interest.

## Authors’ contributions

SF carried out the molecular experiments and drafted the main parts of the manuscript. CT performed the work with the rats and northern blotting, WHZ supported us with the engineered heart tissue, RV performed the calcium uptake experiments, TG helped to draft the manuscript, AL participated in the design of the study and of the manuscript, GAJ participated in its design and coordination, HS helped to draft the manuscript, TH coordinated the molecular experiments and the engineered heart tissue model, JW conceived the study, performed the statistical analysis and drafted parts of the manuscript. All authors read and approved the final manuscript.
